# Investigating the Effects of Recycled Aggregate and Mineral Admixtures on the Mechanical Properties and Performance of Concrete

**DOI:** 10.3390/ma16145134

**Published:** 2023-07-21

**Authors:** Amal Fawzy, Ahmed Elshami, Seleem Ahmad

**Affiliations:** 1 Engineering Materials Department, Faculty of Engineering, Zagazig University, Zagazig 44519, Egypt; amalfawzy164@gmail.com; 2 Housing and Building National Research Centre, Giza 11511, Egypt; materialhnbrc@yahoo.com

**Keywords:** concrete, recycle aggregate, microstructure, nanosilica, slag, compressive strength, tensile strength, flexure strength

## Abstract

In this work, the effects of recycled concrete aggregate, modified with mineral admixtures and nanosilica, on the mechanical properties and performance of concrete after curing in tap water for 28 and 90 days were investigated. The compressive (ƒ_c_), indirect tensile (ƒ_t_), and flexural (ƒ_b_) strengths for the cured concrete specimens were measured, and the concrete strength ratios were analyzed. The water and rapid chloride permeabilities were measured. SEM analysis of the microstructure was also performed. The coarse aggregates used were dolomite (control) and recycled concrete aggregate, incorporating different mineral admixtures, including ground, granulated blast slag, granite, and nanosilica. It was found that the slump values of the dolomite concrete decreased compared with recycled aggregate concrete. Compared to the control mix produced with the recycled aggregate, the slump value of the concrete mixes created with the recycled aggregate increased by approximately 11.1% with the addition of binary cementing materials of 1% NS. The results also indicate that the concrete mix containing the recycled aggregate had the highest compressive strength, tensile strength, and flexural strength compared to that of the dolomite aggregate. Regarding the compressive strength, the addition of 1% NS and 15% slag improved the physico-mechanical properties of the recycled aggregate concretes compared to the other mixes after curing in tap water. Compared to the other mixes, the concrete mix containing 1% NS and 15% slag had a comparatively dense and compact microstructure.

## 1. Introduction

Solid waste pollution is currently one of the most pressing issues because of its detrimental effects on the environment, public health, and the global economy. Therefore, it is vital to effectively utilize and recycle various solid wastes for the creation of building materials, etc. [1,2]. The primary binder for modern buildings is Portland cement. With developments in the construction field, it is anticipated that cement use will rise. According to the IEA, one of the strategies for lowering carbon emissions is to replace cement with other cementitious materials [3]. As a result, many studies have substituted industrial waste and byproducts for cement in certain constructions [4,5,6].

According to earlier studies, the addition of industrial wastes like slag, aluminum, and granite to concrete reduces production costs and carbon emissions while also adding new qualities, including improved mechanical capabilities and durability. Many contemporary concrete mixtures are altered using admixtures which enhance the microstructure and lower the concentration of calcium hydroxide produced via cement hydration (CH) by consuming it in a pozzolanic reaction. The fine particle size of the pozzolanic materials found in the concrete mixes creates an abundance of nucleating sites for the precipitation of hydration products. Nanosilica additions improve the mechanical properties (compressive strength) of the resulting cement paste when compared to the addition of microsilica. In the case of nanosilica, the consumption of Ca (OH)_2_ is completely negligible, and nanosilica promotes the growth of Si chains, thereby forming denser structures. Moreover, nanoscale CNS and C-S-H particles fill the voids between hydrates, thus refining the pore size, increasing the complexity of pores, and improving the microstructure of ITZ, contributing to improved impermeability [7,8,9].

Concrete specimens are more homogeneous due to this technique. This results from the reaction between the pozzolanic material’s amorphous silica (SiO_2_) and the calcium hydroxide (CH) created during the cement hydration process. In addition, the refined grains’ physical effects enable dense filling within the cement specimens and decrease the wall effect in the zone where the paste meets the fine and coarse aggregate [10,11,12,13,14]. 

Fly ash (FA) and slag (GBFS), combined with recycled aggregate, were studied and evaluated for use in the creation of concrete [15,16].

Compressive strength, rupture modulus, and shrinkage tests were carried out as part of an experimental program that manufactured concrete using the substitutes of recycled coarse aggregate with ratios of 0%, 50%, and 100%; slag (GBFS) with ratios of 0% and 50%; and fly ash (FA) with ratios 0% and 50%. According to the study’s findings, the addition of more recycled aggregate boosted the concrete’s strength. The quality of the recycled material, as well as the mixing technique, may be related to this performance. High-quality recycled concrete may be produced by mixing recycled coarse aggregates, fly ash, and slag [17,18,19].

Several studies [20,21] examined the strength of normal concrete produced with the partial replacement of natural coarse particles from demolished waste concrete and the manufacturing of sustainable concrete using various types of waste [22,23,24,25].

The impact of using leftover ceramic aggregate in concrete, both coarse and fine, on the strength of concrete was investigated. According to the findings, the concrete produced using ceramic aggregate waste exhibited an enhanced flexural strength, compressive strength, STS, and elastic modulus. [26]. Equal amounts of recycled and conventional aggregates were employed. Marble dust was used in place of cement in increments of 2.5%, ranging from 0 to 10%. By replacing 5% of the cement with marble dust, the compressive strength was increased by 8.3%, while the weight was reduced by 2% when compared to the results for ordinary concrete. Therefore, it was determined that the ideal dosage of marble dust in green concrete is a 5% replacement rate. Granite waste can be used in place of cement and fine aggregate to increase the strength of concrete. A total replacement of 30% was shown to be the most advantageous quantity. The use of granite powder will prevent disposal issues and eliminate related environmental challenges. Additionally, by using granite waste, less river sand will be used, protecting natural resources [27,28].

Aluminum dross (e.g., aluminum oxide (Al_2_O_3_) and salts) is composed of a mixture of free metal and non-metal materials. Along with metal oxides produced from the molten alloy, aluminum nitrides and carbides may also be present [29]. The 5%, 10%, and 15% replacement levels can produce high-quality concrete. It has been shown that aluminum waste possesses pozzolanic qualities, decelerating the setting times for concrete and thus making it potentially useful for hot-weather concreting. Due to its potential for usage in environmentally friendly concrete constructions, the prospective use of recycled aggregate made from construction and demolition debris has drawn increasing interest. Additionally, the scarcity of natural coarse and fine aggregates in some regions of the world necessitates the development of recycled coarse and fine aggregates as a substitute source of aggregates [30].

The concrete industry can positively impact the environment through the recycling of construction debris, reducing demand for landfill space, conserving resources, saving energy, and reducing pollution, as academics have suggested [2,31,32,33,34]. According to experiments [35,36] performed on concrete samples created by completely swapping out the original aggregates for recycled aggregates that contained a mineral admixture of fly ash (FA), slag (BFS), and silica fume (SF), concrete properties can be developed to meet requirements with the correct mineral selection and proportioning. Recent years have seen a growing interest in nanotechnology for cement research due to the superior physical and mechanical properties of nanomaterials [37,38,39,40]. Different nanoparticles have been effectively used in concrete to enhance its mechanical properties and great durability. Cement pastes have been effectively reinforced using silica nanoparticles, giving them good mechanical properties. The surrounding aggressive environment often leads to the deterioration of concrete structures. In a marine setting, chloride ion (Cl^−^) entered concrete through water or sea winds carrying salt ions that eroded the concrete [40,41,42,43,44,45].

This study thoroughly examined the impacts of recycled aggregates and mineral admixtures (nanosilica fume, granite, slag, and aluminum waste) on the physical and mechanical properties of concrete specimens. We utilized typical values for the cement replacement levels in concrete mixes: 15% slag, 10% granite, 1.5% aluminum waste, and 1% nanosilica. The aim of this research was to produce sustainable concrete and lower carbon emissions using local waste materials in Egypt, such as slag, granite powder, nanosilica, and aluminum waste as a replacement for cement and recycled aggregate from old concrete as a replacement for coarse aggregate (dolomite).

## 2. Materials and Methods

**Figure 1:**
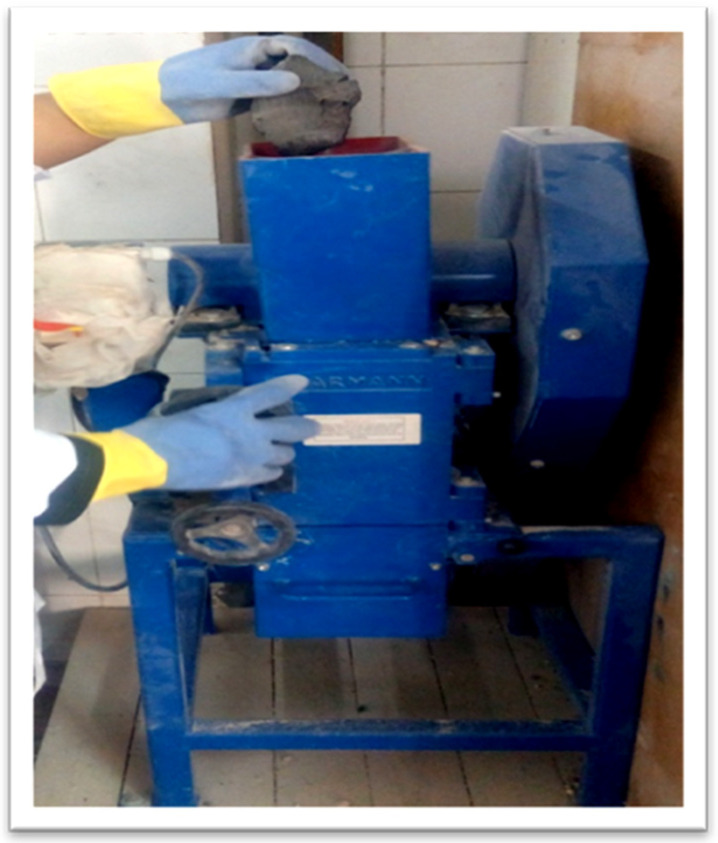
The crushing machine for preparing the recycled coarse aggregate.

**Figure 2:**
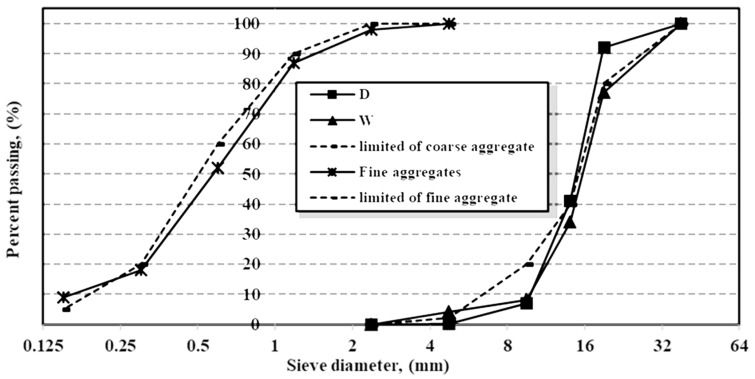
Sieve analysis results for coarse and fine aggregates.

Portland cement CEM1-42.5 N, supplied by the Suez cement business in Suez, Egypt, was used in this work. The used cement met the Egyptian Standard Specifications (4756-1/2007). The mechanical and physical properties of the used cement were confirmed through laboratory testing at the Housing and Building National Research Center (as per E.S.S No. 2421/2005), which indicated that it was suitable for concrete work. The measured properties of the cement used in this study are presented in Table 1. The Iron and Steel Factory in Helwan, Egypt, also produces granulated blast furnace slag (GBFS). The chemical parameters of the raw materials used were confirmed through laboratory testing at the Housing and Building National Research Center, as shown in Table 2, along with several other raw materials like granite dust, aluminum dross, and nanosilica fumes. The nanosilica utilized in this research as a mineral admixture was obtained from the Housing and Building National Research Center in Egypt. The current study’s aggregates were a control coarse aggregate, crushed dolomite, D, and a coarse aggregate from the Attaka area of Suez, Egypt. The recycled concrete aggregates (RCA), W, were obtained from the materials labs of the Housing and Building National Research Center in Egypt. The old concrete specimens were crushed to the required sizes, as shown in Figure 1. According to (ESS 1109 and ASTM C637), the coarse aggregates were manually sieved into size fractions of 5–20 mm. Local sand was cleaned on-site to remove harmful substances, and chloride (Cl^−^), as a contaminant, served as the fine aggregate. Figure 2 shows the grading curves for the aggregates. Table 3 lists the chemical compositions of the aggregates, as confirmed through laboratory testing at the Housing and Building National Research Center. According to the limits outlined by the ESS 1109 and ASTM C637, the coarse aggregates’ physico-mechanical characteristics and fine fractions were assessed. The results are displayed in Table 4. The addition of a superplasticizer allows for a significant reduction in the amount of mixing water while maintaining high workability. A superplasticizer was used to keep the droop at 10 ± 2 cm throughout the experiments. Table 5 shows the concrete mix compositions.

(1) (Egyptian Standard Specification No. 1109 2002).
(2) (Egyptian Code of Practice for Reinforced Concrete No. 203 2017).
(3) (ASTM C637 2009).

A 180 kg pan mixer was used to combine the aggregates, binder material, superplasticizer, and water in order to create concrete. The mixing process involved combining the aggregates for approximately 2 min, adding half the water volume in 15 s, combining the ingredients for 3 min, distributing the binder material and superplasticizer over the aggregate in the pan, and mixing for 30 s. The remaining water was added within the next 30 s after restarting the mixing process. Due to the high surface energy of nanoparticles, they are mixed in a number of stages [46]. After all the ingredients were added, the concrete mix was stirred for 3 min. To ensure uniformity, the mixture was repeatedly tipped into the pan mixer after stirring.

**Figure 3:**
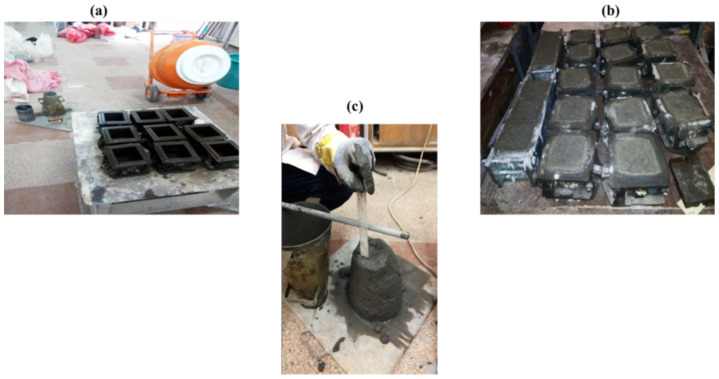
(**a**) Pan mixer and cubic steel molds (10 × 10 × 10 cm). (**b**) Filling the molds. (**c**) Slump test.

Fresh mixtures were slump-tested for workability, Figure 3c, in accordance with (ASTM C143), and all the samples were then cast into molds by roughly layering and compacting them on a vibrating table, as shown in Figure 3b. Immediately after casting, the concrete samples were wrapped in plastic and left in the lab for (24 h) at (23 ± 2 °C) and 100% relative humidity. The specimens were submerged in water after demolding until the time for testing.

**Figure 4:**
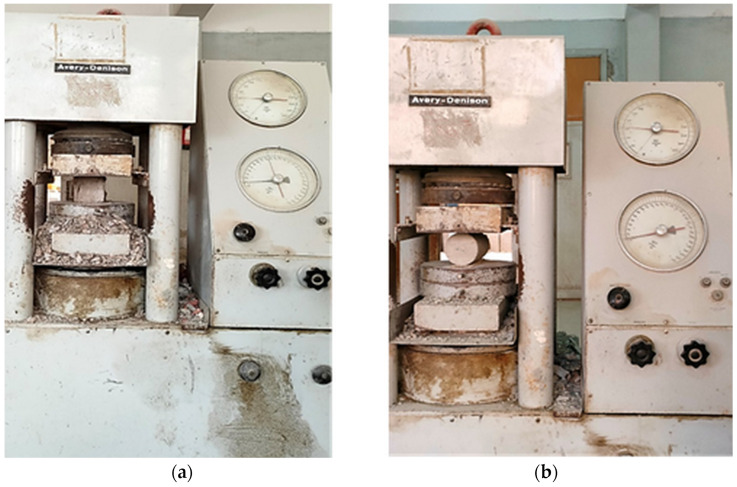
Test setup. (**a**) Compression test. (**b**) Splitting tensile test.

According to ASTM C511, the cubes were cured and subjected to strength tests at 28 and 90 days, as per EN 196-1:2016. A 2500 kN Avery Denison machine, as shown in Figure 4, with a compliant loading rate of 1 kN/min was used for the compressive and indirect tensile strength tests of three samples per test, canceling any results deviating from the mean by 25%, according to the Egyptian specifications. 

**Figure 5:**
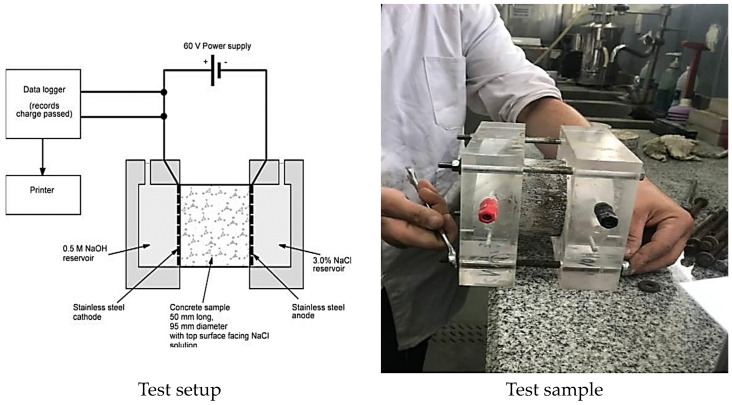
Rapid chloride permeability testing (RCPT).

**Figure 6:**
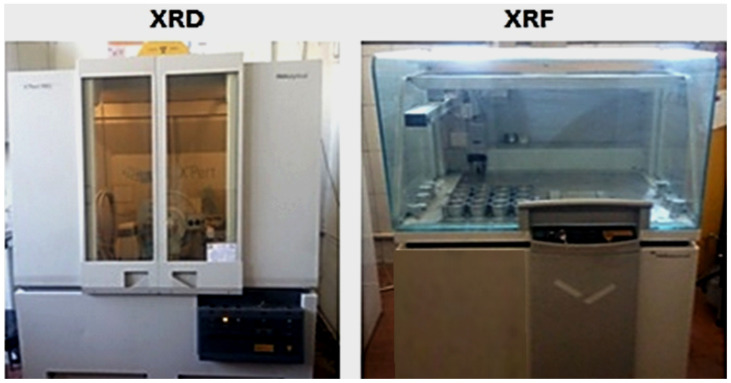
X-ray diffraction (XRD) and X-ray fluorescence (XRF) apparatus.

**Figure 7:**
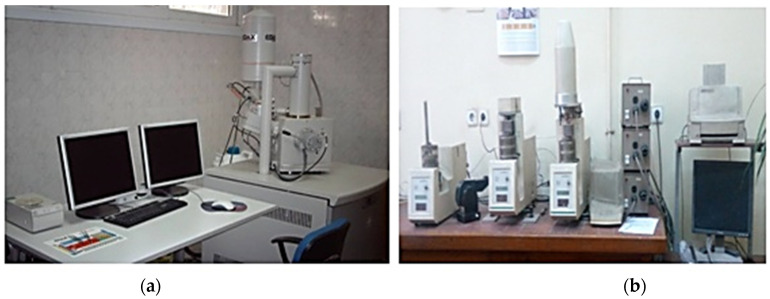
(**a**) Scanning electron microscope (SEM) and (**b**) thermo-gravimetric analyzer (TGA).

ASTM C1202 states that a cylindrical specimen of 50 × 100 mm is submerged in a 30% NaCl solution for a chloride permeability test, as shown in Figure 5. One end of the specimen is submerged in 0.3 M NaOH, while a steady voltage of 60 V is delivered across the specimen’s ends for a measuring period of 6 h. The electrical current that flows through the sample is measured in Coulombs. The starting materials were analyzed using an XRF Spectrometer Philips PW1400, as shown in Figure 6. The samples were prepared with a Rb-kα radiation tube at 50 kV and 50 mA. Pellets were finely ground to pass through a 75 µm sieve and pressed at 20 tons. The tested samples were collected from the innermost cores of the crushed specimens after the compressive strength test at 28 days. The test was conducted at Egypt’s Housing and Building National Research Center. Scanning electron microphotographs were taken for the selected samples using an Inspect (S) apparatus (FEI Company, Eindhoven, Holland) equipped with an energy-dispersive X-ray analyzer (EDAX) Figure 7. The microscope can examine the microstructure of a fractured composite at an accelerating voltage of 200 V to 30 kV and power zoom magnification up to 300,000 ×.

Samples from the chosen specimens were taken, processed, and tested under TGA/DTG. Testing was performed on the selected samples of dried paste using a TGA-50 thermal analyzer. The analysis was carried out after completing a compression test at the age of 28 days. A sample of approximately 50 mg (−53 µm) was used with a heating rate of 20 °C/min up to 1000 °C in a dynamic nitrogen atmosphere. The TGA investigations revealed the decomposition of the cement hydrates through diminishing increments on the TGA curves and endothermic peaks on the derivative TGA. Figure 7 shows the test apparatus.

## 3. Results and Discussions

### 3.1. Workability

**Figure 8:**
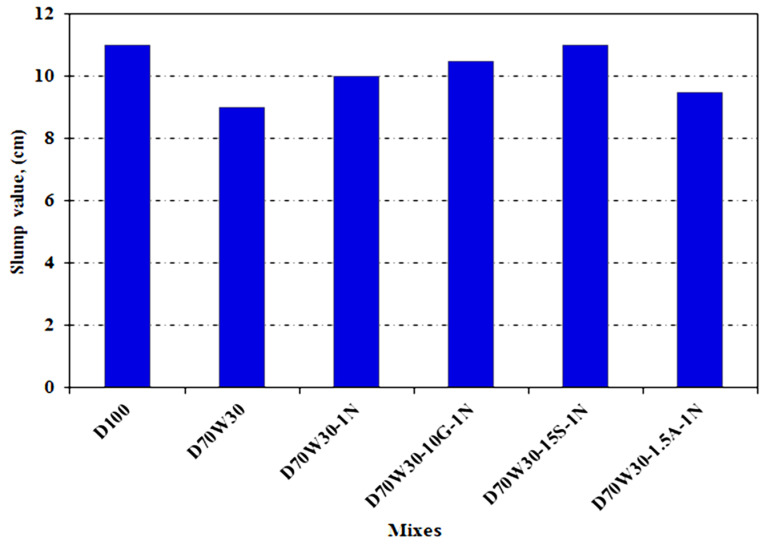
Slump values for concrete mixes (cm).

The slump test is the simplest way to determine workability. Figure 8 shows that almost all the combinations had a droop of 8–11 cm. The data in Figure 8 indicate the slump of fresh mixes containing dolomite aggregate, the recycled concrete aggregate D70W30, and mixes containing recycled concrete aggregate mixed with the following: 1% nanosilica and nanosilica, D70W30-1N; 1% nanosilica, nanosilica, and 10% granite, D70W30-1N-10G; 1% nanosilica, nanosilica fume, and 15% blast furnace slag, D70W30-1N-15S; and 1% nanosilica, nanosilica, and 1.5 aluminum waste, D70W30-1N-1.5. Concrete formed with dolomite aggregate, slump = 11 cm, performs better than concrete including recycled aggregate, slump = 9 cm, in terms of workability, according to the results. This is because recycled aggregate is more porous and has a higher water absorption value than dolomite aggregate, as shown in Table 4. The results also show that, in comparison to the control mix produced with the recycled aggregate, slump = 9 cm, the slump value of the concrete mixes created with recycled aggregate increased with the addition of binary cementing materials containing the following: 1% NS and 10% granite, slump = 10.5 cm; 1% NS and 15% slag, slump = 11 cm; and 1% NS and 1.5% aluminum waste, slump = 9.75 cm. Due to its smoother form and spherical structure, OPC requires less water when slag, granite, and nanosilica are added to the mix. The addition OF aluminum waste to concrete mixtures reduces THE slump value compared to mixes with 1% NS and 10% granite or 1% NS and 15% slag due to aluminum powder’s high water absorption value. [40,45].

### 3.2. Compressive Strength

**Figure 9:**
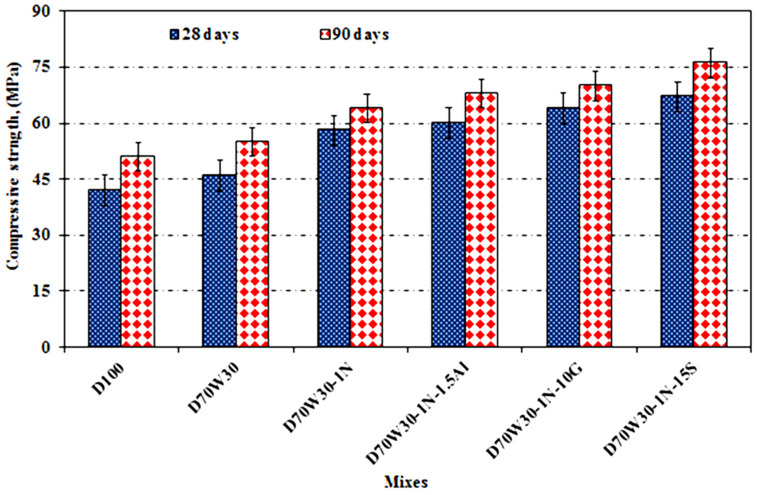
Compressive strength of concrete mixes after curing in tap water for up to 90 days.

In Figure 9, the compressive strengths of concrete mixes containing dolomite and recycled concrete aggregate with various mineral admixtures as partial replacements for OPC are shown graphically. All the mixes were cured in tap water. The results indicate that the strength values increase with the curing time due to the reduced porosity and increased content of binding materials. In the concrete with recycled aggregates, the effective w/c is no lower than that of the concrete without. The compressive strength is higher in the former case due to the hydration process and increased amount of cement hydration products that were deposited in the open-pore system of the hardened concrete. The concrete produced with recycled aggregate has a better compressive strength than the concrete constructed with dolomite aggregate due to the improved interlocking between their porous textures and cement paste. Similar results were reported in [25,32,40]. In contrast, the results show that the compressive strength values increased with the addition of nanosilica by 26.1%, in the case of D70W30-1N, and the binary cementing materials by 39.2%, 45.6%, and 30.9%, in the case of D70W30-1N-10G, D70W30-1N-15S, and D70W30-1N-1.5A, respectively, at the age of 28 days. This was mainly caused by the concentration and fineness of the amorphous silica, nanosilica, granite, slag, and cementing materials, which have a filler effect and pozzolanic reaction that refine pores. The pozzolanic reaction of lime, which precipitates in open pores to produce a more compact, closed structure, converts lime formed from the hydration of cement into additional binding components.

### 3.3. Tensile Splitting Strength

**Figure 10:**
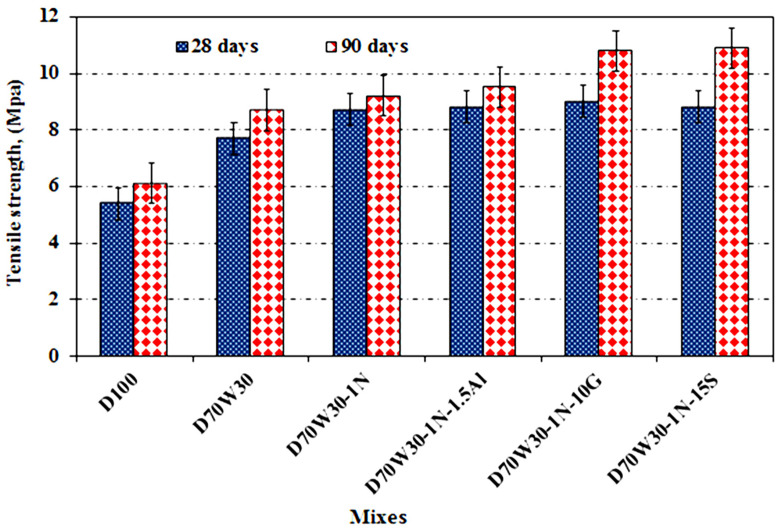
Tensile strength of concrete mixes after curing in tap water for up to 90 days.

The tensile strength values of the concrete mixes were evaluated at days 28 and 90. Figure 10 illustrates the results. The tensile splitting strength of the recycled aggregate concrete and the recycled aggregate concrete incorporating mineral admixtures of nanosilica, granite, slag, and aluminum waste was higher than that of the control concrete mixes at all ages. The splitting tensile strength of the concrete mixes formed with the recycled aggregate, D70W30, and recycled aggregate containing nanosilica, D70W30-1N, as well as the binary cementing materials of D70W30-1N-10G, D70W30-1N-15S, and D70W30-1N-1.5A, all increased significantly compared to the control concrete at 28 days of age. The greatest improvements were observed in concrete mixes made with the recycled aggregate D70W30 and NS, slag, granite, and aluminum waste as mineral admixtures, which enhanced the microstructure of the interfacial transition zone (ITZ) and strengthened the binding between the cement and aggregate in the concrete mixes.

### 3.4. Flexural Strength

**Figure 11:**
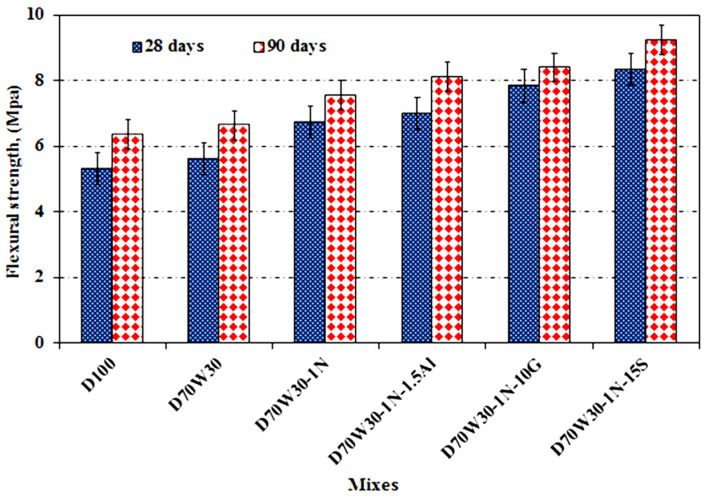
Flexural strength of concrete mixes after curing in tap water for up to 90 days.

Figure 11 shows outcomes for the flexural strength of the concrete mixes on days 28 and 90. The flexural strength increases with the curing time, as does the compressive strength for all combinations. The recycled aggregate concrete and recycled aggregate concrete with mineral admixtures of nanosilica, granite, slag, and aluminum waste had higher flexural strength than the equivalent control concrete mixtures at all test ages. The flexural strength of the mixes made with recycled aggregate, D70W30, and aggregate containing nanosilica, D70W30-1N, as well as the binary cementing materials D70W30-1N-10G, D70W30-1N-15S, and D70W30-1N-1.5A, increased by 5.3%, 26.3%, 47.2%, 56.7%, and 31.5%, respectively, at the age of 28 days, compared to the control concrete, D100.

### 3.5. Strength Ratios

**Figure 12:**
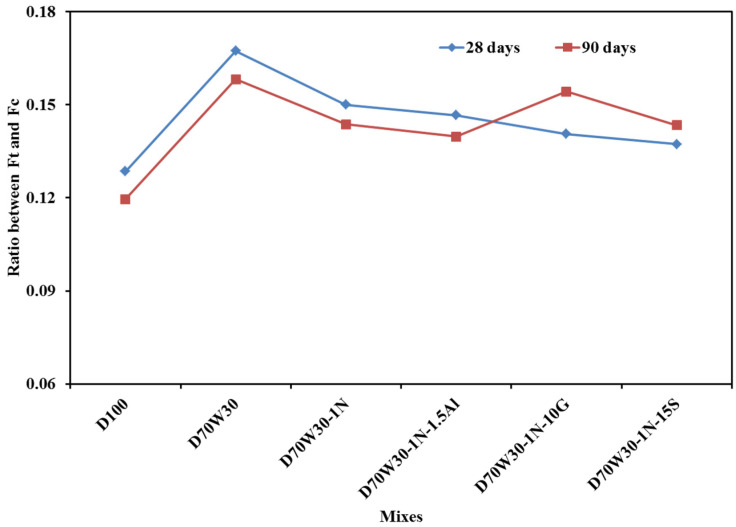
The ratio between tensile strength and compressive strength.

**Figure 13:**
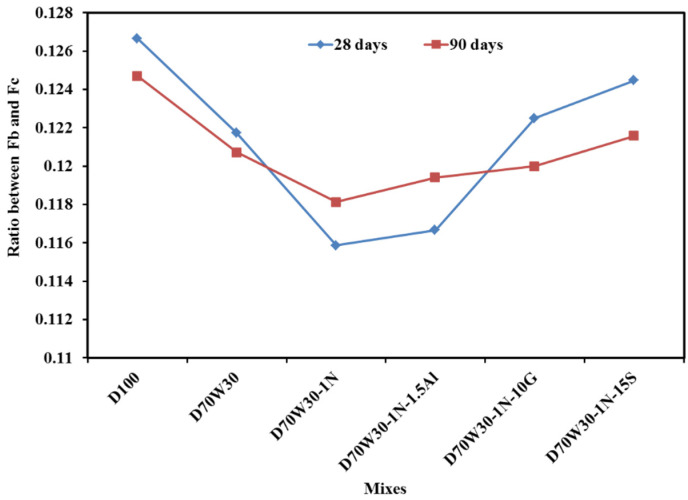
The ratio between flexural strength and compressive strength.

Figure 12 displays the strength ratio between the indirect tensile strength and compressive strength for all mixes at 28 and 90 days. D70W30, the recycled aggregate mix, has a higher strength ratio than the control mix, D100, due to an increase in its indirect tensile strength at 28 and 90 days, while the other mixes show a slightly decreasing ratio, though still higher than the control mix. In contrast, Figure 13 shows that the flexural strength ratio remains higher for the control mix, D100, which may be attributed to changes in the enhancement rate of the compressive, indirect tensile, and flexural strength resulting from the use of the recycled aggregate and mineral admixtures.

### 3.6. Water Permeability

**Figure 14:**
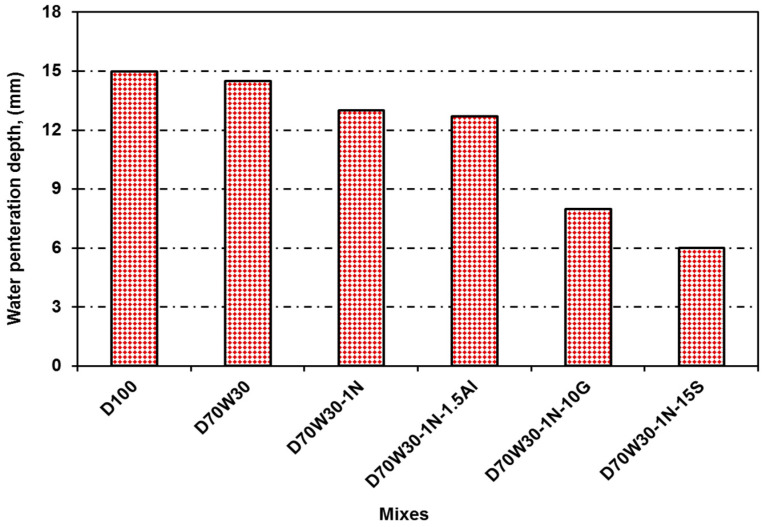
The impact of concrete type on concrete’s ability to absorb water.

The depth of water penetration under pressure indirectly determines water permeability. Figure 14 shows the impact of concrete type on water absorption. The recycled aggregate concrete and recycled aggregate concrete with mineral admixtures of nanosilica, granite, slag, and aluminum waste reduced the water permeability of the concrete compared to the control concrete mixtures. At 28 days of age, the concrete mixes containing the recycled aggregates D70W30 and 70W30-1N, as well as the binary cementing materials D70W30-1N-10G, D70W30-1N-15S, and D70W30-1N-1.5A, showed decreases in water permeability of 3%, 13%, 46%, 60%, and 15%, respectively, compared to the control concrete, D100. The mixing water was absorbed by the aggregates, reducing the quantity of efficient water, workability, and the water–cement ratio, thus reducing water absorption.

### 3.7. Rapid Chloride Permeability

**Figure 15:**
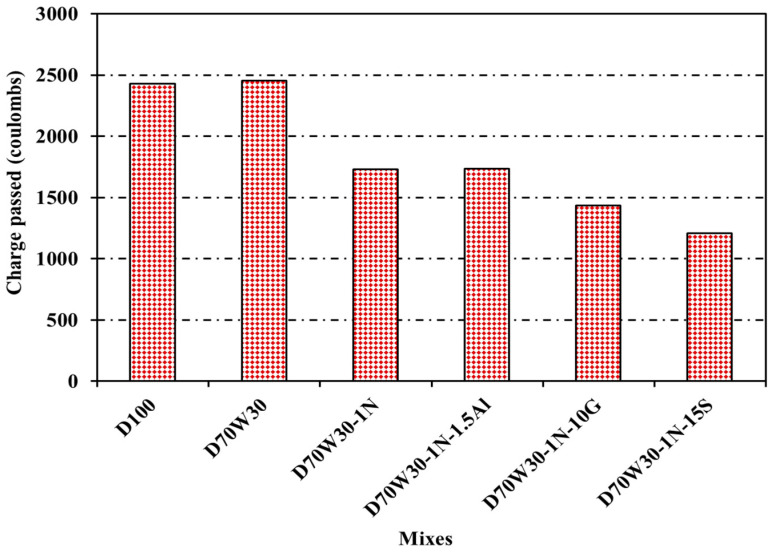
Chloride penetration resistance of concrete mixes.

According to ASTM C1202-C97, the cylinder specimens underwent RCPT, and after 6 h of applying 60 volts, the Coulombs representing the total charge passed was observed. Figure 15 displays the measured values and standard deviations. At 28 days old, the concrete control and the recycled aggregate version, D70W30, displayed moderate chloride permeability, with values of 2431 and 2454 Coulombs, respectively. These values were reduced to 1732, 1739, 1439, and 1210 Coulombs for the used concrete comprising recycled aggregate containing nanosilica, D70W30-1N, and the binary cementing materials of D70W30-1N-1.5A, D70W30-1N-10G, and D70W30-1N-15S, respectively, which may be characterized as "low to moderate." The findings indicated a decrease in the total charge transmitted with the additional mineral admixtures, which might have enhanced the interfacial transition zone’s (ITZ) microstructure and strengthened the link between the cement and the aggregate, resulting in a relatively dense and compact microstructure. A similar finding was reported in [47], where the results showed that the 28 d chloride ion migration coefficient of concrete with different admixtures was generally reduced with the increase in the proportion of fly ash and grinding blast furnace slag.

### 3.8. TGA/DTG Analysis

**Figure 16:**
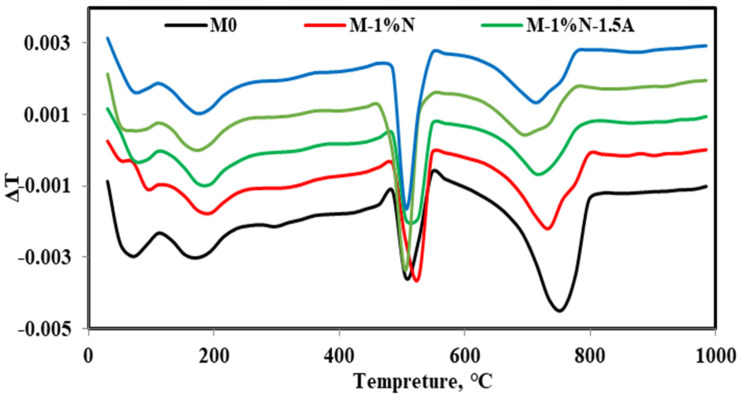
DTG of cement specimens after curing in tap water at 28 days.

**Figure 17:**
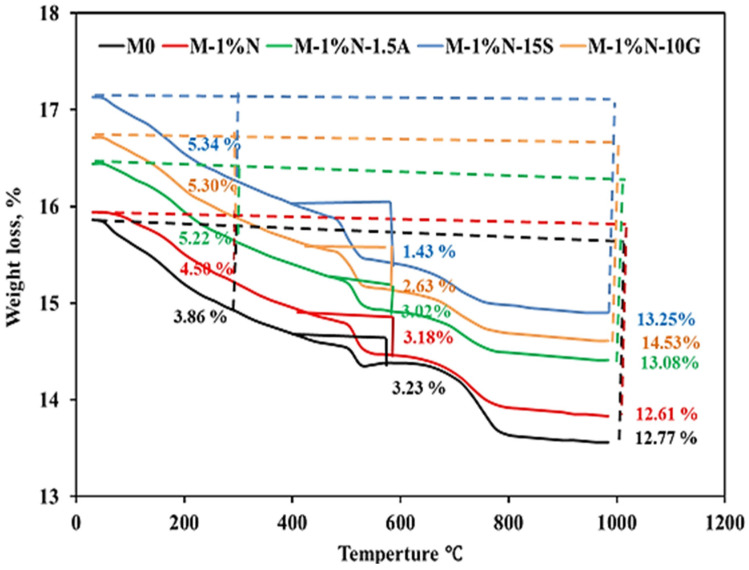
Thermo-gravimetric analysis of cement specimens after curing in tap water at 28 days.

All chosen samples underwent evaluation at temperatures ranging from 25 °C to 1000 °C. After 28 days of hydration, the TGA/DTG curves of OPC, OPC-1N, OPC-1N-1.5Al, OPC-1N-10G, and OPC-1N-15S were obtained, as displayed in Figure 16 and Figure 17. The majority of the samples underwent weight loss below 300 °C, followed by losses at 500 °C and then below 800 °C. The dehydration of free water and C-S-H gel is said to be responsible for weight loss below 300 °C, which corresponds to the first main endothermic peak. The OPC-1N-15S samples have the lowest peak intensity at 500 °C, while the weight loss results for the samples of OPC, OPC-1N, OPC-1N-1.5Al, OPC-1N-10G, and OPC-1N-15S are 3.23%, 3.18%, 3.02%, 2.63%, and 1.43%, respectively, implying an increase in C-S-H gel and a decrease in Ca (OH)_2_ generation.

### 3.9. Microstructure Analysis

**Figure 18:**
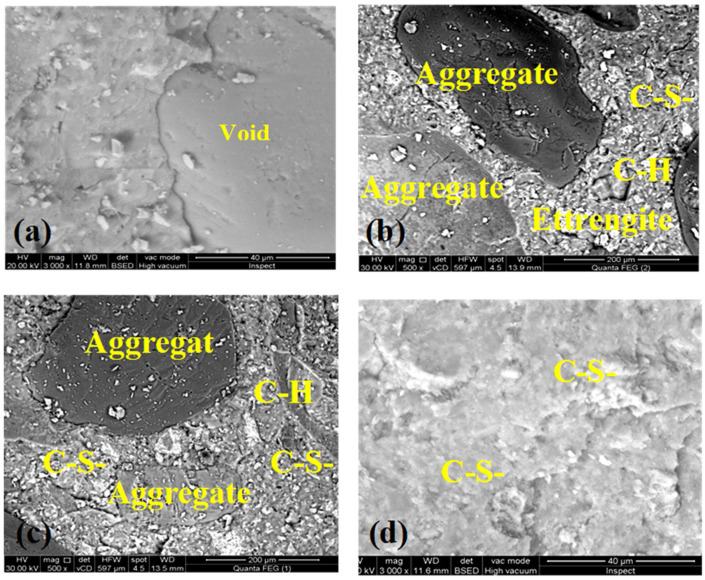
SEM images of the concrete mixes (**a**) D100, (**b**) D70W30, (**c**) D70W30-1N, and (**d**) D70W30-15S-1N at the age of 28 days.

Figure 18 shows SEM images of the concrete mixes D100, D70W30, D70W30-1N, and D70W30-15S-1N after curing for 28 days in tap water. The micrographs demonstrate a compact microstructure attributed to the increased C-S-H phase and hydration products. D70W30-15S-1N has a denser and more compact microstructure due to the high pozzolanic activity of the nanosilica and slag, resulting in the formation of additional C-S-H gel deposited in the pores throughout the concrete matrix.

## 4. Conclusions

This paper presented an experimental study on the performance of recycled aggregates and mineral admixtures with respect to their mechanical and microstructural properties. The study investigated the materials’ compressive, flexural, and tensile strengths, chloride penetration, and microstructure. The findings provide fundamental knowledge, as follows:

(1) The slump value of dolomite concrete decreases as compared to recycled aggregate concrete. However, the addition of 1% NS binary cementing material increases the slump value of the concrete mix produced with recycled aggregate by approximately 11.1%.
(2) The concrete mix with recycled aggregate has higher compressive, tensile, and flexural strength than dolomite aggregate because the recycled aggregate’s physical and mechanical characteristics improve upon interlocking with cement paste.
(3) The strength increase is paired with a decrease in chloride penetration, indicating that microcracking affects both chloride penetration and compressive strength.
(4) The inclusion of nanosilica and mineral admixtures in recycled aggregate concrete improves its mechanical strength.
(5) The micrographs show that concrete mixes with recycled aggregate and mineral admixtures have a more compact microstructure compared to normal dolomite concrete due to an increased C-S-H phase and hydration products.

## Figures and Tables

**Table 1:** Physical and mechanical properties of the used cement.

Test		Test Results	E.S.S Limits
Specific gravity		3.15	-------
Specific surface area (cm^2^/gm)		3290	≥2750
Setting time (min)	Initial	110	≥60 min
	Final	380	≤10 h
Compressive strength 3 days (MPa)		20.6	≥10 MPa
Compressive strength 28 days (MPa)		50.1	≥42.5 MPa

**Table 2:** Chemical composition of OPC, NS, granite, and slag (wt. %).

Oxide Content, (%)	OPC	Nanosilica	Granite	Slag	Aluminum Waste
SiO_2_	21.33	95.22	69.50	36.66	16.40
AL_2_O_3_	3.99	0.32	14.50	10.32	75.90
Fe_2_O_3_	3.15	0.85	3.01	0.52	1.97
CaO	62.04	0.26	3.0	38.88	1.64
MgO	2.52	0.55	0.64	1.70	1.01
SO_3_	2.70	0.20	0.19	2.16	0.12
L.O.I.	3.75	1.50	0.65	0.13	0.10
Na_2_O	0.26	0.37	3.46	0.45	-
K20	0.22	0.51	4.29	1.02	-
Cl^−^	0.01	0.05	0.11	0.05	0.05
TiO_2_	-	-	0.37	0.56	-
TOTAL	99.95	99.98	99.99	99.97	99.61
Ins. Res	0.66	-	-	-	-
Na_2_OEq.	0.41	-	-	-	-
L.S.F	0.90	-	-	-	-
C3A	5.22	-	-	-	-
C3S	51.40	-	-	-	-
C2S	22.47	-	-	-	-
C4AF	9.59	-	-	-	-

**Table 3:** Chemical compositions of the starting materials (wt. %).

Oxides, (%)	Dolomite	Recycled Dolomite Aggregate	Sand
SiO_2_	1.67	19.10	93.40
Al_2_O_3_	0.07	2.45	2.03
Fe_2_O_3_	0.01	1.50	0.98
CaO	35.54	37.61	0.71
MgO	17.51	8.87	0.25
SO_3_	0.13	1.51	0.30
Cl	-	0.13	0.08
Na_2_O	0.04	0.49	0.38
K_2_O	0.02	0.16	0.64
TiO_2_	0.01	0.19	0.17
BaO	-	-	-
P_2_O_5_	0.01	-	0.06
MnO	-	-	0.03
(L.O.I)	44.99	27.83	0.74
Total	99.97	99.97	99.92

**Table 4:** Physical and mechanical properties of coarse aggregates and their fine portions.

Physical & Mechanical Properties	Sand	Coarse Aggregates		Limits of Coarse Aggregates
		Dolomite	Recycled Dolomite Aggregate	
Specific gravity (g/cm^3^)	2.65	2.61	2.41	-
Unit weight (ton/m^3^)	1.62	1.46	1.37	-
Clay and fine materials (%)	1.40	0.38	-	≤4(1) ≤10(3)
Water absorption (%)	-	1.09	4.49	≤2.5(1)
Flakiness index (%)	-	15.12	18.70	≤25(2)
Elongation index (%)	-	13.79	9.20	≤25(2)
Abrasion resistance (%)	-	19.32	25.84	≤30(2)
Impact value (%)	-	15.52	19.31	≤45(1)

**Table 5:** Mix proportions for concrete per (1 m^3^).

Composition	Concrete Ingredients (kg/m^3^)									
	OPC	Slag	Granite	Aluminum Waste	Nanosilica	W	Fine Aggregates	Coarse Aggregates		SP
							Sand	D	W	
D100	450	-	-	-	-	173	772	1544	-	8.1
D70W30	450	-	-	-	-	173	772	1081	463	9.7
D70W30-IN	445.5	-	-	-	4.5	173	772	1081	463	9.5
D70W30-1.5A-1N	438.75	-	-	6.75	4.5	173	772	1081	463	9.6
D70W30-10G-1N	400.5	-	45	-	4.5	173	772	1081	463	9.4
70W30-15S-1N	378	67.5	-	-	4.5	173	772	1081	463	9.1

## Data Availability

Not applicable.
